# Application of dual-energy CT in assessing the efficacy of nasopharyngeal carcinoma and diagnosing metastatic lymph nodes

**DOI:** 10.3389/fonc.2025.1602222

**Published:** 2025-11-27

**Authors:** Yunnong Wang, Feng Dai, Yang Li, Wei Qiao, Kang Li, Chunhan Pan, Qianlai Jiang, Shanyu Fang, Zheng Kang, Xiuming Zhang

**Affiliations:** 1 Jiangsu Cancer Hospital, Nanjing Medical University, Nanjing, China; 2 Nanjing Medical University, Nanjing, Jiangsu, China; 3 Intervention Department, Nanjing Second Hospital, Nanjing, Jiangsu, China

**Keywords:** dual-energy CT, multi-parameters, nasopharyngeal carcinoma, diagnostic accuracy, metastatic lymph nodes

## Abstract

**Purpose:**

To explore the value of dual-energy CT (DECT) in evaluating the efficacy of nasopharyngeal carcinoma (NPC) and in the detection of metastatic lymph nodes (MLNs).

**Materials and methods:**

For this retrospective study, we collected and analyzed clinical and imaging data from 83 patients diagnosed with NPC via histopathology, who were admitted to the Radiotherapy Department from August 2022 to July 2024. The cohort consisted of 64 males and 19 females, with an average age of 50.86 ± 13.45 (years). All patients underwent DECT enhancement and MRI scanning before and after the first treatment course to assess the extent of lesions and lymph nodes (LNs). A total of 423 LN imaging datasets were analyzed. We measured iodine concentration (IC), effective atomic number (Zeff), electron density (ED), and normalized iodine concentration (NIC). Additionally, we recorded the magnetic resonance ADC values of the LNs.

**Results:**

Statistical analysis of Zeff, ED, and NIC values of lesion and LNs revealed no significant differences between groups (*p* > 0.05). The Zeff, ED, and NIC values for lesions and LNs were significantly lower after treatment than before (*p* < 0.05). Receiver operating characteristic (ROC) analysis for MLNs indicated that the area under the ROC curve (AUC) for NIC indicated high accuracy.

**Conclusion:**

DECT provides valuable functional parameters for assessing the efficacy of NPC and demonstrates significant clinical application value. Notably, the NIC parameter shows high diagnostic efficacy for MLNs, comparable to the ADC value obtained from MRI.

## 1 Introduction

Nasopharyngeal carcinoma (NPC) is a malignant epithelial tumor that originates in the nasopharynx and exhibits a high geographical prevalence (1). Dual-energy CT (DECT) has demonstrated superior performance in diagnosing skull base invasion in NPC (2). Its short scanning time allows for 1-mm thin-layer reconstructions, providing an advantage in visualizing fine structures. Additionally, DECT facilitates material characterization and differentiation, as well as the calculation of virtual monoenergetic reconstructions, which can enhance lesion detection and delineation (3, 4).

Traditionally, the standard assessment of tumor response to treatment is determined by the RECIST 1.1 criteria, which primarily focus on dimensional changes of the tumor. However, with the widespread use of antiangiogenic therapies (e.g. monoclonal antibodies), relying solely on changes in lesion size is inadequate as an indicator of treatment response (5). DECT enables the estimation of iodine concentration (IC) in tissues through iodine-water material decomposition, allowing for a more objective assessment of tumor volume delineation, vascular characteristics, and overall tumor burden (6). Thus, we propose utilizing the relevant parametric properties of DECT to address the limitations of the RECIST criteria.

NPC is associated with a high incidence of lymph node (LN) metastasis, with cervical LN metastasis rates exceeding 70%. This significantly affects both clinical staging and treatment strategies and is a crucial factor influencing prognosis (7). Currently, the identification of metastatic LNs in clinical practice primarily relies on measuring the maximum short diameter of LNs and assessing features such as necrosis. However, a standardized diagnostic criterion for differentiating between metastatic and non-metastatic LNs is lacking. Therefore, this study aims to explore the diagnostic efficacy of DECT parameters in distinguishing metastatic from non-metastatic LNs.

## 2 Materials and methods

### 2.1 Patients

**Figure 1:**
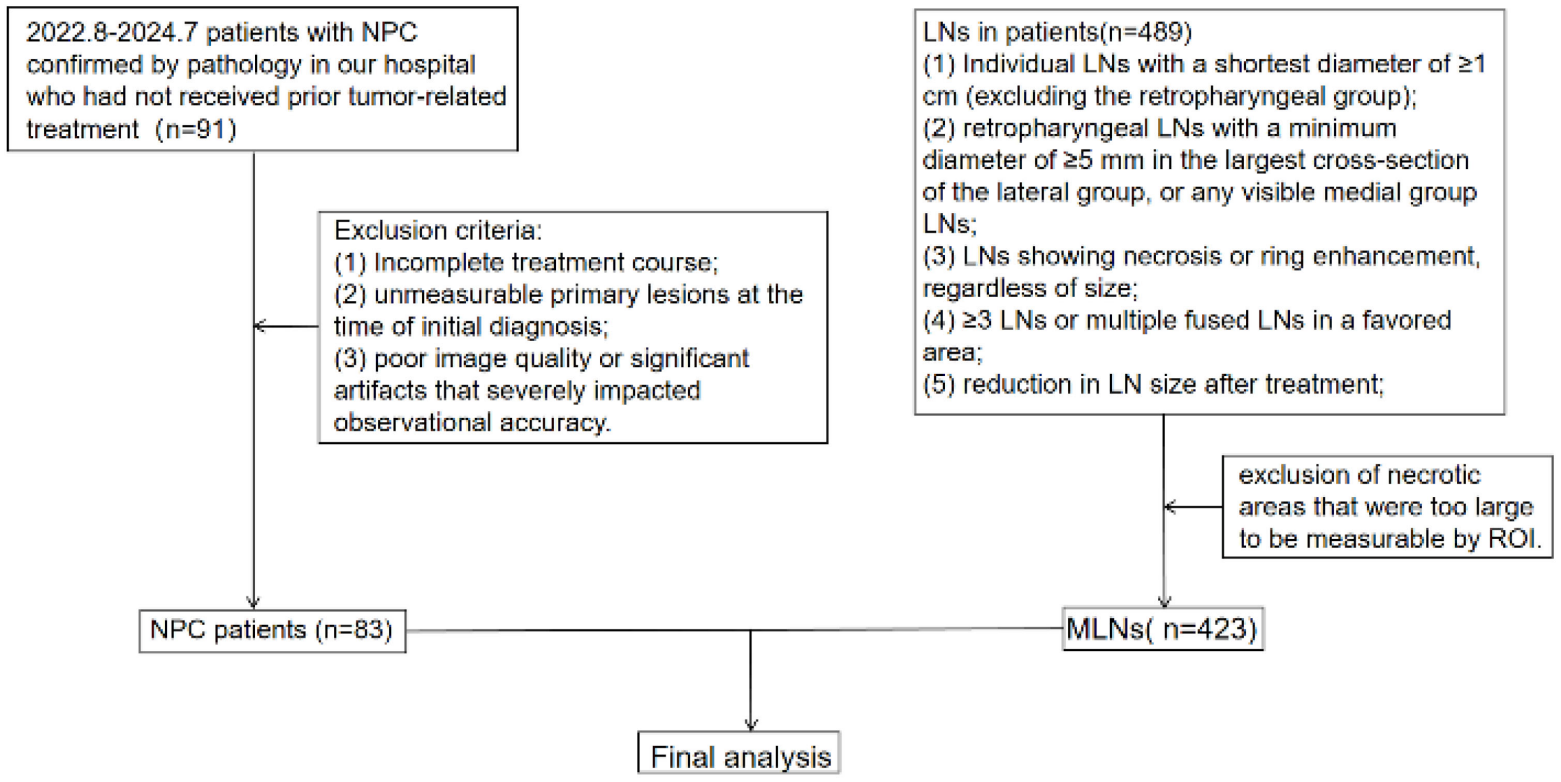
Flowchart showing patient inclusion and exclusion in the study.

We retrospectively analyzed clinical and imaging data from 83 patients with NPC who were admitted to the Radiotherapy Department between August 2022 and July 2024. All patients were pathologically diagnosed and underwent comprehensive clinical and imaging evaluations (**Figure 1**). All patients underwent concurrent chemoradiotherapy (CCRT), among these patients, 22 patients had received targeted therapy and 39 patients had received anti-angiogenic drug therapy. The cohort consisted of 64 males and 19 females, with an average age of 50.86 ± 13.45 (years). Due to the non-specific early symptoms of NPC, many patients were already in advanced stages at the time of diagnosis. As a result, the 83 patients were categorized into three groups: T1 and T2 (18 cases), T3 (29 cases), and T4 (36 cases; **Table 1**). After treatment, patients were further divided based on the RECIST 1.1 evaluation criteria into three groups: effective, stable, and progression. The effective group included individuals who achieved complete remission (CR) or partial remission (PR), whereas the stable group encompassed patients with stable disease (SD). No patients were classified in the progression group. In total, 44 patients were classified into the effective group and 39 into the stable group. All patients underwent DECT enhancement and MRI scans before treatment and at the end of the first treatment course, with efficacy subsequently evaluated. This study was approved by our institutional ethics committee(No. 2020-055), which waived the requirement for informed consent. All personal information was anonymized during the study.

**Table 1:** Baseline characteristics of 83 patients.

Characteristic	No. patients	%
Age(yrs)	50.86 ± 13.45	
Gender		
Male	64	77.1
Female	19	22.9
T classifications		
T1	14	16.9
T2	4	4.8
T3	29	34.9
T4	36	43.4
TNM stage		
I	0	0
II	2	2.4
III	22	26.5
IV a	53	63.9
IV b	6	7.2

Inclusion criteria: (1) Patients with primary NPC who had not received prior tumor-related treatment; (2) availability of complete clinical and imaging data before and after treatment; and (3) absence of contraindications to treatment. Exclusion criteria: (1) Incomplete treatment course; (2) unmeasurable primary lesions at the time of initial diagnosis; and (3) poor image quality or significant artifacts that severely impacted observational accuracy.

### 2.2 Dual-energy spectral CT imaging

In this study, a clinical DECT scanner (IQon, Philips Healthcare, Best, The Netherlands) was employed. A non-ionic contrast agent (iophorol 350 g/ml) was administered intravenously via the median forearm vein at a rate of 2.5 mL/s. Scanning parameters were as follows: collimation of 64 × 0.625 mm, rotation time of 0.5 s, pitch of 0.671, tube voltage of 50 keV, and tube current of 182 mA. The scanning range extended from the maxillary sinus to the thoracic inlet, and images were acquired 35 s after contrast injection to capture enhanced images.

The scanned images were reconstructed at 50 keV monoenergetic levels, producing axial, coronal, and sagittal thin-layer reconstructions, each with a slice thickness and interval of 1.0 mm. DECT images, including multi-energy monoenergetic maps (MonoE), iodine maps (Iodine no Water), and effective spectral images, were generated. Two experienced radiologists independently processed these reconstructions using the Philips IntelliSpace Portal software.

### 2.3 Images analysis

We assessed the boundaries and extent of NPC lesions. Referring to the diagnostic imaging criteria for MLNs in the neck (8), we selected positive LNs based on the following criteria: (1) Individual LNs with a shortest diameter of ≥1 cm (excluding the retropharyngeal group); (2) retropharyngeal LNs with a minimum diameter of ≥5 mm in the largest cross-section of the lateral group, or any visible medial group LNs; (3) LNs showing necrosis or ring enhancement, regardless of size; (4) ≥3 LNs or multiple fused LNs in a favored area; (5) reduction in LN size after treatment; and (6) exclusion of necrotic areas that were too large to be measurable by ROI. A total of 423 LNs were collected from the initial imaging data of the 83 NPC patients, comprising 293 metastatic and 130 non-metastatic LNs, based on the established criteria for cervical LN metastasis.

**Figure 2:**
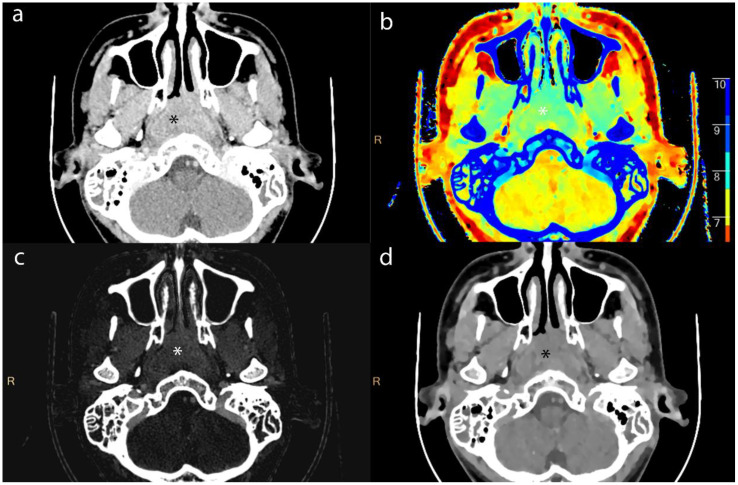
Axial contrast-enhanced CT images of lesion (*) from a 16-year-old male patient with NPC. **(a)** shows the short-axis diameter and CT value of the lesion. **(b)**- **(d)** shows the Zeff maps, Iodine no water maps and ED maps of lesion.

**Figure 3:**
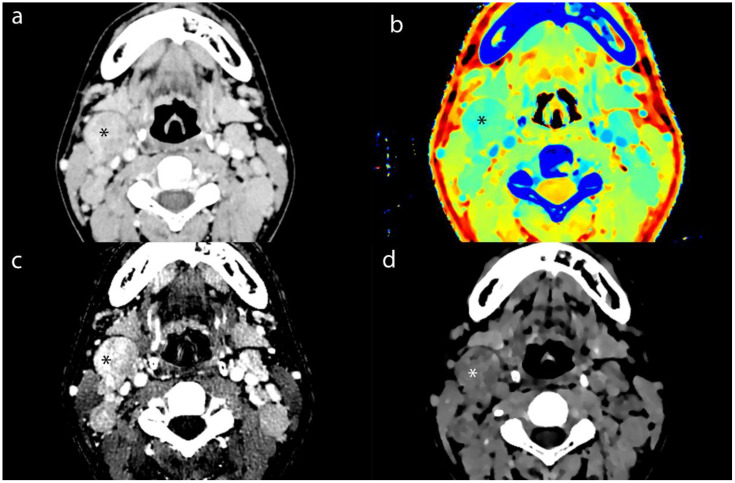
Axial contrast-enhanced CT images of MLN (*) from a 16-year-old male patient with NPC. **(a)** shows the short-axis diameter and CT value of the MLN. **(b)**- **(d)** shows the Zeff maps, Iodine no water maps and ED maps of MLN.

**Figure 4:**
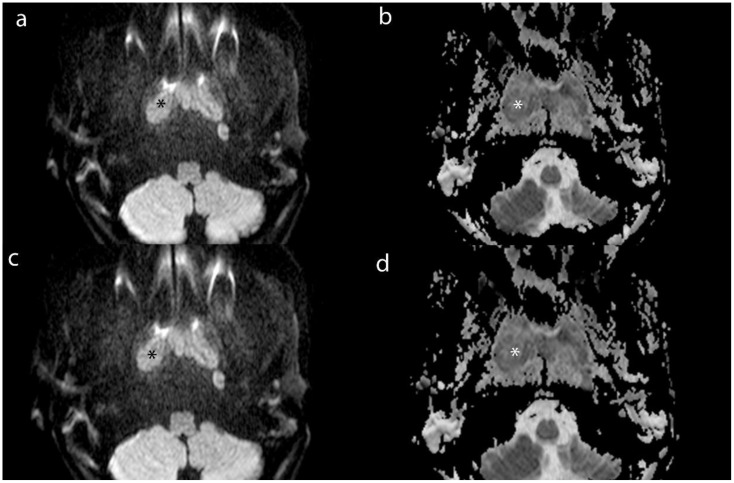
Axial MR images of lesion (*) and MLN (*) from a 16-year-old male patient with NPC. **(a)** and **(b)** show the DWI and ADC images of lesion. **(c)** and **(d)** show the DWI and ADC images of MLN.

We measuring the largest cross-section, and recorded the CT value, IC, effective atomic number, and electron density (ED) of enhanced tumor lesions and positive LNs, alongside the ICs of the internal jugular artery and vein. And we also measured the ADC values of tumor lesions and positive LNs on MRI To avoid contamination from necrotic areas, we selected three regions of interest (ROI), each approximately 1 cm in diameter, manually avoiding tumor margins by 2 mm. The average of these measurements was calculated (**Figures 2**–**4**).

### 2.4 Statistical analysis

Statistical analyses were performed using the SPSS and Original software package. All measurement data were expressed as mean ± standard deviation (¯*x* ± *s*). Normality tests were conducted on the parameter values of lesions and positive LNs before and after treatment in both the effective and stable groups. For normally distributed data, paired-sample *t*-tests were utilized, while the Mann-Whitney U test was applied to non-normally distributed data. Intra- and inter-rater agreement between the measurements recorded by the two observers were assessed using the intraclass correlation coefficient (ICC). ICC values were interpreted as follows: poor reliability: <0.5; moderate reliability: 0.5–0.75; good reliability: 0.75–0.9; excellent reliability: >0.90. Group means were compared using analysis of variance (ANOVA). A *p*-value of <0.05 was considered statistically significant. The diagnostic efficacy of each energy spectrum parameter was assessed using receiver operating characteristic (ROC) curve analysis, with diagnostic performance quantified by the area under the curve (AUC; AUC = 0.5-0.7 indicating low accuracy; AUC = 0.7-0.9 indicating moderate accuracy; AUC > 0.9 indicating high accuracy). The diagnostic accuracy of DECT parameters and ADC were evaluated by comparing the cutoff value we analyzed. For each comparison, a confusion matrix and histogram we used to calculate accuracy estimate. The accuracy, precision, recall, and F1 score were calculated.

## 3 Results

### 3.1 Intergroup analysis of lesions

**Figure 5:**
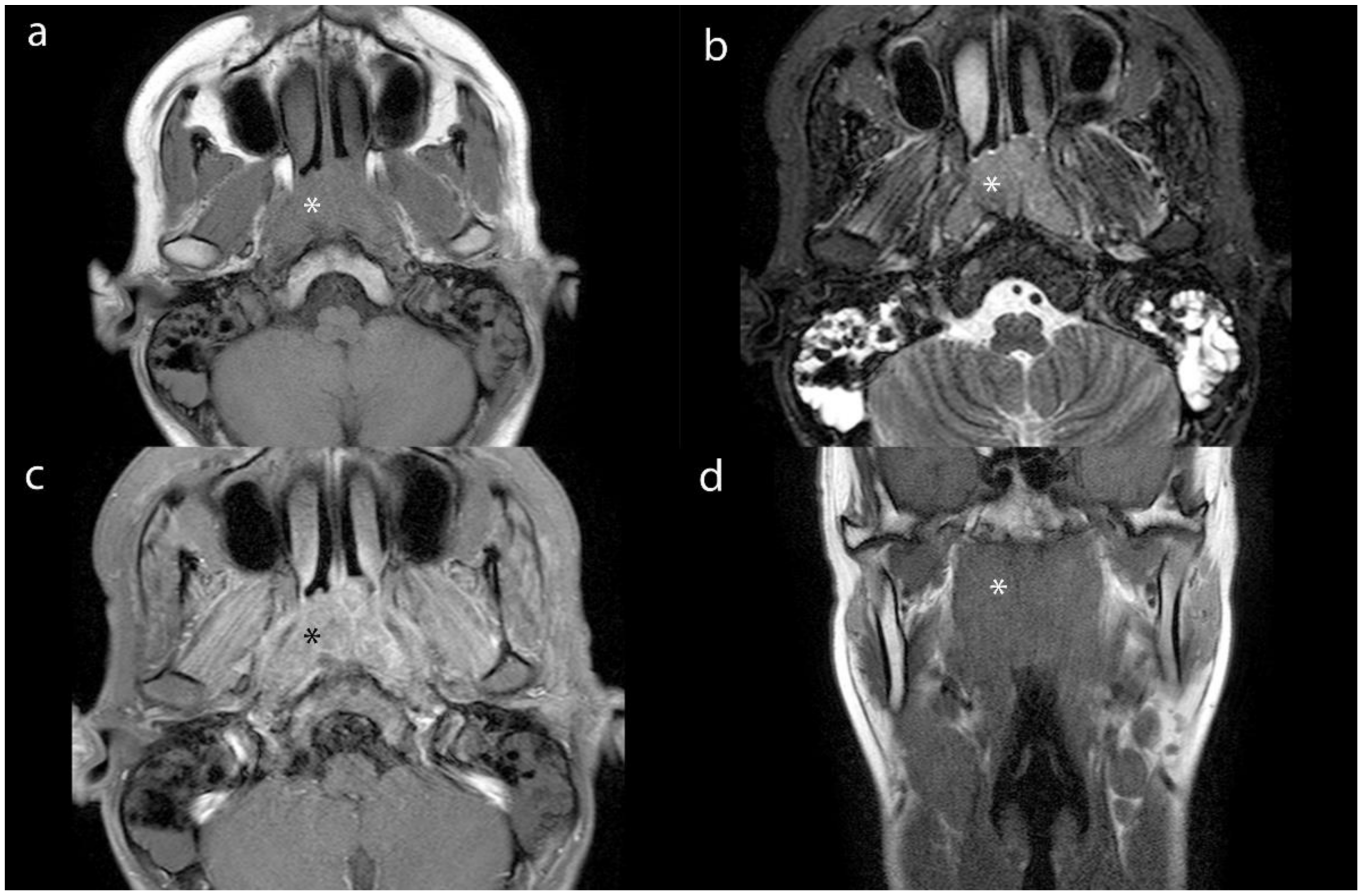
Axial MR images **(a-c)** and coronal MR image **(d)** of lesion (*) from a 16-year-old male patient with NPC. **(a)**, **(b)** and **(c)** show the T1WI, STIR and contrast- enhanced images. **(d)** shows the T1WI image.

The *t*-test analysis shows that the effective atomic coefficients, electron cloud density values, and NIC values of lesions among the three different groups of NPC were 0.24 (8.37± 0.29, 8.42± 0.24 and 8.50± 0.27), 0.22 (105.02± 0.83, 104.68± 0.70 and 104.73± 0.5) and 0.88 (0.42± 0.16, 0.42± 0.14 and 0.40± 0.12), respectively, the values of MLNs were 0.94 (8.37± 0.19, 8.38± 0.25 and 8.38± 0.20), 0.14 (104.58± 0.52, 104.32± 0.41 and 104.33± 0.51) and 0.34 (0.43± 0.13, 0.40± 0.14 and 0.38± 0.10); the differences in effective atomic coefficients, electron cloud density values, and NIC values of lesions and LNs among the three different staging groups of NPC were not statistically significant (*p* > 0.05). However, DECT multiparametric imaging effectively demonstrated the extent of lesions, showing a high degree of consistency when compared with corresponding MRI scans. In certain cases, DECT provided superior delineation of subtle structures at the skull base compared to MRI (**Figure 5**).

The measurements made by the two radiologists were consistent, demonstrating strong interobserver agreement. All ICC values > 0.750. The ICC values of quantitative variables for inter-observer measurements ranged from 0.813 (95% CI: 0.726–0.875) to 0.996 (95% CI: 0.994–0.997).

The maximum diameter of lesions and LNs in the 83 patients decreased after treatment compared to pre-treatment measurements. Among these patients, 44 in the effective group achieved either CR or PR, while the 39 patients in the stable group did not meet the criteria for CR or PR. Overall, the parameter values for lesions and LNs were significantly reduced post-treatment, with statistically significant changes observed in all parameters (p < 0.05; **Tables 2**, **3**).

**Table 2:** Changes in parameters before and after treatment in the effective group.

Parameters	Group		*P* Value
	Before	After	
Lesions			
Zeff	8.47 ± 0.29	6.38 ± 3.50	0.00
NIC	0.39 ± 0.13	0.23 ± 0.16	0.00
ED (%)	104.81 ± 0.78	80.62 ± 44.23	0.01
Short-axis diameter (cm)	3.38 ± 1.28	2.17 ± 0.82	0.00
MLNs			
Zeff	8.33 ± 0.19	6.38 ± 3.51	0.01
NIC	0.35 ± 0.12	0.23 ± 0.16	0.00
ED (%)	104.33 ± 0.54	80.50 ± 44.16	0.01
Short-axis diameter (cm)	2.07 ± 1.12	1.37 ± 0.54	0.00

**Table 3:** Changes in parameters before and after treatment in the stable group.

Parameters	Group		*P* Value
	Before	After	
Lesions			
Zeff	8.41 ± 0.23	8.15 ± 0.28	0.00
NIC	0.44 ± 0.13	0.27 ± 0.12	0.00
ED (%)	104.74 ± 0.55	104.48 ± 0.46	0.00
Short-axis diameter (cm)	3.63 ± 1.31	2.55 ± 1.07	0.00
MLNs			
Zeff	8.15 ± 0.28	8.22 ± 0.24	0.00
NIC	0.45 ± 0.12	0.29 ± 0.10	0.00
ED (%)	104.43 ± 0.13	104.29 ± 0.49	0.04
Short-axis diameter (cm)	2.68 ± 1.12	1.99 ± 0.73	0.00

### 3.2 Quantitative analysis of metastatic LNs

**Figure 6:**
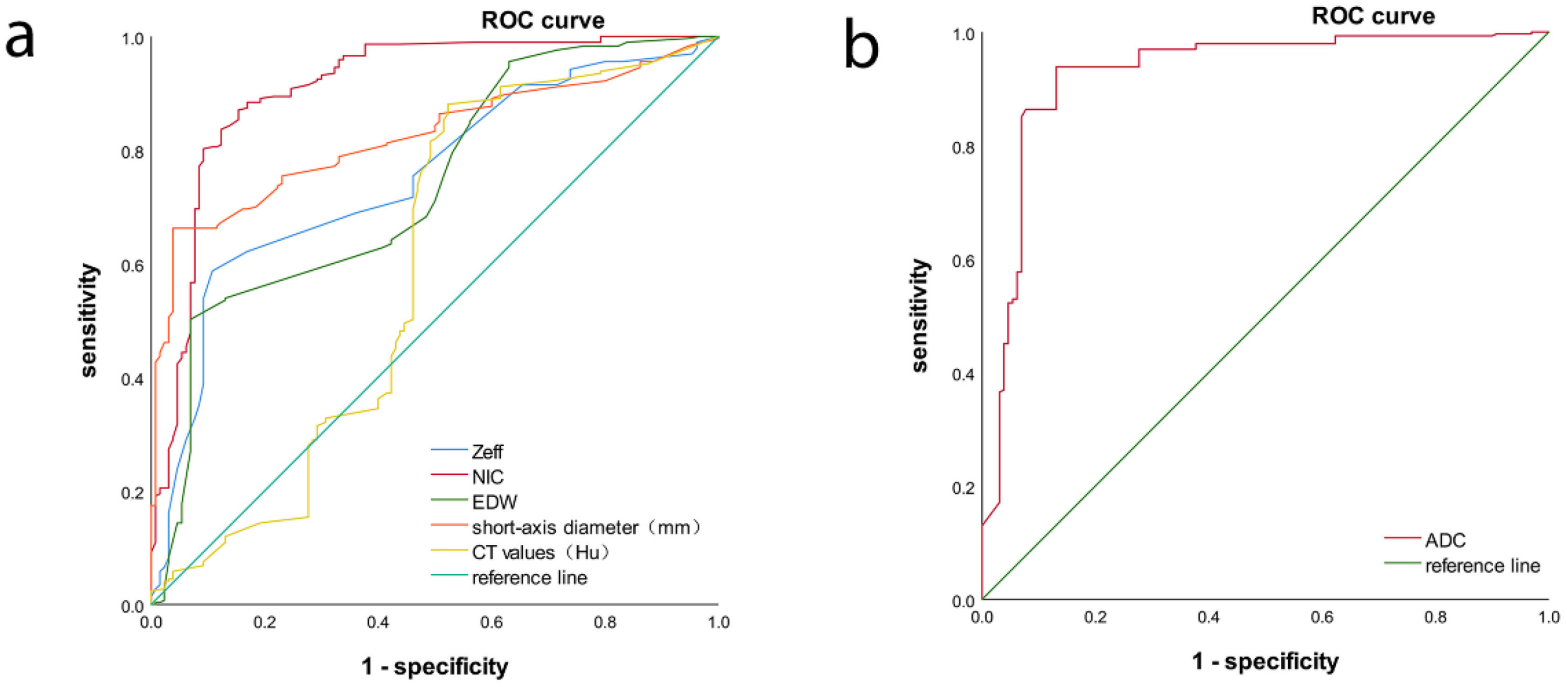
ROC of MLNs, **(a)** is from DECT parameters, **(b)** is from ADC value.

**Figure 7:**
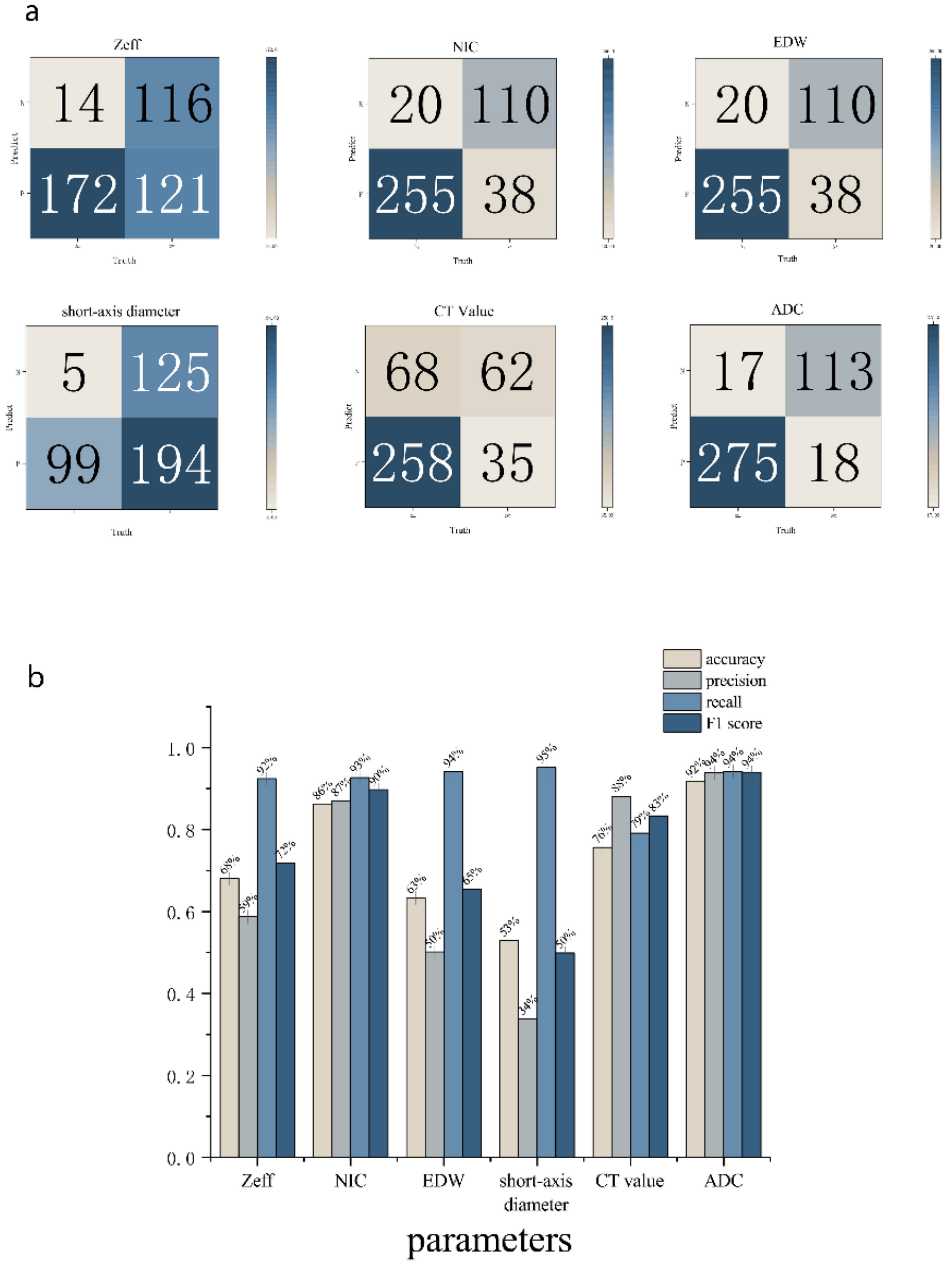
Confusion Matrix and histogram of results of DECT parameters **(a)** and ADC **(b)**.

ROC analysis indicated that the AUC for Zeff, NIC, ED maximum short diameter, CT values, and magnetic resonance ADC values of the LNs were 0.751 (95% CI, 0.702-0.800), 0.910 (95% CI, 0.875-0.944), 0.735 (95% CI, 0.684-0.787), 0.822 (95% CI, 0.783-0.861), 0.590 (95% CI, 0.522-0.657), and 0.9270 (95% CI, 0.894-0.959), respectively. The corresponding sensitivities were 58.7%, 87.0%, 50.2%, 66.2%, 88.1%, and 93.9%, whereas the specificities were 89.2%, 84.9%, 93.1%, 96.2%, 47.7%, and 86.9% (**Table 4**, **Figures 6**, **7**). These results indicate that CT values had limited accuracy as a diagnostic criterion for metastatic LNs, whereas Zeff and ED demonstrated moderate accuracy. In contrast, NIC, similar to the maximal short diameter and the ADC value from MRI, exhibited high accuracy.

**Table 4:** ROC of MLNs.

Quantitative parameters	Cutoff value	AUC	Sensitivity(%)	Specificity(%)	*P* values
Zeff	8.45	0.751	58.7	89.2	0.00
NIC	0.63	0.901	87.0	84.6	0.00
ED (%)	104.14	0.735	50.2	93.1	0.00
Short-axis diameter(cm)	2.54	0.822	66.2	96.2	0.00
CT values(Hu)	99.70	0.590	88.1	47.7	0.03
ADC(10^-3^×mm^2^/s)	0.80	0.920	93.9	86.9	0.00

## 4 Discussion

NPC is notably sensitive to radiotherapy, making it the preferred treatment option. Accurate delineation of the target zone is crucial for effective radical treatment and for minimizing the risk of recurrence and metastasis. This necessitates thorough assessment of tumor invasion using CT and MRI (9). NPC is also prone to early LN metastasis, with the number and distribution of metastatic LNs playing a critical role in staging, treatment planning, and prognosis (10). Furthermore, precise segmentation of metastatic LNs is essential for effective radiotherapy planning (11).

Imaging evaluation is vital throughout the radiotherapy process for NPC. However, traditional assessments may not sufficiently capture tumor response in a timely and sensitive manner (12). DECT generates multiple sequence images through material separation, providing a more intuitive representation of substance distribution within tissues. This technique enables the assessment of substance concentrations, such as iodine-water matrix separation (13). DECT offers a more accurate depiction of tumor blood supply than conventional enhancement scans, as the heterogeneity of IC images correlates with intra-tumor perfusion and microcirculatory variability (14). Thus, DECT effectively addresses the limitations of assessing tumors solely based on their maximum diameter.

In this study, patients who had undergone chemotherapy, targeted therapy, or immunotherapy prior to radiotherapy were selected. DECT was used to evaluate these patients both before treatment and after one course of neoadjuvant therapy. We compared and assessed changes in lesion parameters among different staging groups and before and after treatment in 83 patients with NPC. Additionally, we analyzed quantitative parameters of cervical LNs using DECT sequences, including effective atomic number maps, IC maps, and electron cloud density maps. Our results showed no statistically significant differences in these parameters between different staging groups, indicating that, despite varying tumor sizes and degrees of invasion before treatment, there was no significant change in tumor heterogeneity.

The Zeff, NIC, and ED values in the effective and stable groups after treatment were significantly lower than those before treatment (*p* < 0.05). This suggests that, in addition to morphological regression and improvement of NPC lesions after adjuvant treatment, there were significant changes in functional parameters such as tumor blood supply. These findings align with previous research (12, 15). The NIC parameters, in particular, facilitate the diagnosis of metastatic LNs and help differentiate them from non-metastatic LNs based on the IC profile (16).

Due to the inherent mechanisms of treatment, lesions may exhibit various phenotypic changes, such as necrosis and reduced blood supply, alongside size reduction. Therefore, assessing treatment efficacy based solely on morphological dimensions is insufficient and may lead to underestimation (17). Previous studies have shown a strong correlation between IC values from DECT analysis and vascular density of lesions (5). Thus, DECT imaging can serve as an early predictor of treatment efficacy, regardless of lesion regression. Additionally, the application of NIC helps mitigate the limitations of relying solely on maximum short diameter for diagnosing metastatic LNs.

In our study, we observed that IC, Zeff, and ED values of lesions and LNs decreased after treatment compared to pre-treatment levels. The images generated using material decomposition techniques allowed for quantification and characterization of relevant tissue components. Iodine maps were generated by subtracting the aqueous component from contrast-enhanced CT images. These maps are instrumental in differentiating enhancing from non-enhancing lesions, reflecting angiogenesis, and visualizing the blood supply to tumor lesions (18). The iodine map significantly enhances the visibility of lesions and helps delineate tumor margins, facilitating the detection of microscopic lesions and tumors with subtle morphological changes. Studies have demonstrated that iodine maps improve the detectability of gastric and colorectal tumors, as well as the distinction between benign and malignant lesions (19, 20). Additionally, a reduction in IC within tumor foci and metastatic LNs post-treatment indicates a decrease in neoplastic capillaries.

The effective atomic number map reflects the average atomic number of tissue compounds, which can be enhanced by increased IC. This map is more sensitive to trace iodine than conventional CT values measured in Hounsfield units, as it is based on ED (19). Elevated Zeff values in pre-treatment lesions and LNs may result from two factors: increased penetration of the iodine contrast agent due to the dense network of neoplastic capillaries and the higher nuclear mass of closely packed tumor cells, which can also contribute to elevated Zeff values (21).

An electron cloud density map illustrates the probability of an electron being present at a specific location, depending on the type and structure of the molecule (22). The primary advantage of electron cloud density mapping is that it can be derived directly from CT images. Since it reflects cell density, it provides valuable insights into tissue composition (23, 24). DECT offers more accurate ED values compared to conventional CT, with ED values expressed as a percentage relative to water’s ED (%EDW). While ED maps are clinically applied in radiotherapy for traditional dose calculations, their use in diagnostic imaging remains limited. although they provided lower contrast in soft tissues, but they are more effective for evaluating disease in very dense or very low-density organs (25).

Our experiment revealed no significant differences in CT values between metastatic and non-metastatic LNs. Although enhanced CT images can partially reflect vascular distribution, they do not necessarily correlate with the degree of enhancement. Moreover, CT values on contrast-enhanced images are influenced by baseline values from non-contrast scans (26). Evaluated CT values of abdominal LNs also showed overlap between metastatic and non-metastatic cases. Thus, CT values may offer limited utility in distinguishing metastatic from non-metastatic LNs in NPC. Traditionally, LN metastasis is assessed based on size; however, our study indicates that relying solely on size is insufficient for accurate differentiation. While larger LNs are often metastatic, smaller LNs can also harbor metastases. In these smaller LNs, the functional parameters obtained through DECT may better reflect tissue blood volume and vascular permeability. These functional parameters demonstrated diagnostic value in identifying metastatic LNs, with NIC values in DECT approaching the accuracy of MRI for this purpose. Thus, dual-energy CT proves valuable in diagnosing metastatic LNs (27).

The limitations of this study include a relatively small sample size. Future clinical research should involve larger cohorts to further investigate the application of DECT parameters in the diagnosis and prognosis of NPC. Additionally, the criteria for identifying metastatic LNs were primarily based on imaging diagnostic standards rather than confirmation through pathological biopsy.

In summary, dual-energy spectral CT advances beyond traditional RECIST criteria, which primarily focus on tumor morphology, by enabling functional imaging capable of detecting internal changes within tumors. Quantitative analysis of parameters derived from effective atomic number maps, electron cloud density maps, and iodine-water maps provides insight into the tumor’s microcirculatory state. These functional parameters facilitate the evaluation of treatment efficacy in both NPC lesions and LNs, guiding subsequent therapeutic strategies. Moreover, pseudo-color images generated from Zeff maps offer superior delineation of lesion boundaries compared to conventional CT localization images. Looking ahead, integrating Zeff maps with localization images holds promise for more precise target delineation, allowing for accurate segmentation of both primary lesions and metastatic LNs.

## Data Availability

The original contributions presented in the study are included in the article/**Supplementary Material**. Further inquiries can be directed to the corresponding author.

## Glossary

DECT: Dual-energy CT
NPC: Nasopharyngeal carcinoma
MLNs: Metastatic lymph nodes
LNs: Lymph nodes
IC: Iodine concentration
Zeff: Effective atomic number
ED: Electron density
NIC: Normalized iodine concentration
ROC: Receiver operating characteristic
AUC: Area under the ROC curve
CR: Complete remission
PR: Partial remission
SD: Stable disease
