# Supplementary material for: Application of dual-energy CT in assessing the efficacy of nasopharyngeal carcinoma and diagnosing metastatic lymph nodes
Source: Front Oncol. 2025 Nov 27;15:1602222. doi: 10.3389/fonc.2025.1602222 (PMC12695558; doi:10.3389/fonc.2025.1602222)
Supplement: Supplementary file 1 [file Table1.docx]

Supplementary table 1.parameters of 3 groups of patients

|  | Zeff | | | EDW | | | NIC | | |  |
| --- | --- | --- | --- | --- | --- | --- | --- | --- | --- | --- |
|  | Lesion | Lymph nodes | | Lesion | Lymph nodes | | Lesion | | Lymph nodes |  |
| T1,T2 group | 8.37±0.29 | | 8.37±0.19 | 105.02±0.83 | | 104.58±0.52 | | 0.42±0.16 | 0.43±0.13 | |
| T3 group | 8.42±0.24 | | 8.38±0.25 | 104.68±0.70 | | 104.32±0.41 | | 0.42±0.14 | 0.40±0.14 | |
| T4 group | 8.50±0.27 | | 8.38±0.20 | 104.73±0.50 | | 104.33±0.51 | | 0.40±0.12 | 0.38±0.10 | |
| *P* value | 0.24 | | 0.94 | 0.22 | | 0.14 | | 0.88 | 0.34 | |
